# Education During Ward Rounds: Systematic Review

**DOI:** 10.2196/40580

**Published:** 2022-11-09

**Authors:** Zahra Khalaf, Shaheer Khan

**Affiliations:** 1 Department of Postgraduate Surgical Studies Royal College of Surgeons in Ireland Dublin Ireland

**Keywords:** education, learning, rounds, trainee, ward rounds, medical education, simulation-based learning, digital health, digital learning, education intervention

## Abstract

**Background:**

Enhancing the educational experience provided by ward rounds requires an understanding of current perceptions of the educational value of rounds.

**Objective:**

This systematic review examines perceptions of education in ward rounds, educational activities in ward rounds, barriers to learning, and perceptions of simulation-based ward rounds.

**Methods:**

The 2020 PRISMA (Preferred Reporting Items for Systematic Reviews and Meta-Analyses) guidelines were followed. MEDLINE (EBSCO), Cochrane, and Scopus were searched on May 29, 2022, for studies assessing learning during ward rounds. The search terms included “ward rounds,” “education,” and “trainees.” Then, the selected articles were reference searched. In total, 354 articles were retrieved. The articles were assessed for eligibility by 2 independent reviewers who screened titles, abstracts, and full-length texts. Articles addressing trainees’ education in all ward rounds were included. Articles were excluded if they were specific to certain disciplines, were reviews, were not published in scholarly journals, were published before 2015, were published in languages other than English, or did not concern human participants. Following the removal of 63 duplicates, a total of 268 articles were excluded. The risk of bias within the selected articles was also assessed via the Critical Appraisal Skills Programme checklist for qualitative research. Qualitative data were used to describe results in a narrative synthesis and in tables.

**Results:**

A total of 23 articles were included. Perceptions of teaching in rounds were addressed by 6 studies, of which 3 showed negative perceptions among participants, 2 reported ambivalent perceptions, and 1 showed positive perceptions. Perceived barriers to teaching during rounds were assessed by 7 studies. The reported barriers included time constraints, workloads, schedules, interruptions, the service-oriented nature of rounds, the lack of feedback, hierarchies, the lack of opportunities to ask questions and be engaged in patient management, and divergent learner needs. Further, 8 studies identified types of educational activities, including observation, patient-specific teaching, and discussion. Perceptions of learning through simulated ward rounds were assessed by 8 studies, and a consensus of satisfaction was noted among learners. The interventions that were explored to improve education included using teaching frameworks, involving clinical librarians, and changing the setting of ward rounds.

**Conclusions:**

The main limitations of this review are the predominant use of qualitative data in the included articles and the lack of standardization for the educational compositions of ward rounds among articles, which made the articles hard to compare. In conclusion, learning opportunities in ward rounds are often missed, and trainees perceive rounds to have low educational value. It is important to recognize the barriers to education during ward rounds and address them to maximize the benefits of ward rounds. Finally, there is a need to develop plans that incorporate teaching regularly during ward rounds in the inpatient setting.

**Trial Registration:**

PROSPERO CRD42022337736; https://www.crd.york.ac.uk/prospero/display_record.php?RecordID=337736

## Introduction

Ward rounds are conducted by teams of health care practitioners to review, assess, and manage patients in an inpatient setting, visiting each patient in order at their bedside. Ward rounds also represent an opportunity for trainees to learn and enhance their clinical and interpersonal skills [1,2]. The educational component of rounds is impacted by workloads, time constraints, and physicians’ teaching attitudes and practices [3]. This has resulted in predominantly negative perceptions of the educational value of ward rounds [4,5]. The recognition of ward rounds as an educational platform has resulted in initiatives, such as simulated ward rounds, that aim to enhance the educational opportunities typically gained through ward rounds.

Enhancing the educational experience provided by ward rounds requires an understanding of current perceptions and experiences. This review examines perceptions of the educational value and content of ward rounds and the interventions that have been explored to optimize education in ward rounds.

## Methods

### Study Design

This systematic review followed the 2020 PRISMA (Preferred Reporting Items for Systematic Reviews and Meta-Analyses) guidelines [6]. This review was registered in PROSPERO (ID number: CRD42022337736).

### Search Strategy

MEDLINE (EBSCO), Cochrane, and Scopus were searched on May 29, 2022, for studies examining the educational value of ward rounds. The search terms used included “ward rounds,” “rounds,” “teaching,” “education,” “learning,” “junior,” and “trainee.” Search limiters were added to narrow the search; the limiters included publications in scholarly (peer-reviewed) journals, English-language articles, human studies, and a date of publication ranging from 2015 to the date of the search. The search was restricted to papers published from 2015 to the date of the search in order to ensure that recent trends in teaching and education in wards were identified. Table 1 shows details on the search terms.

**Table 1:** Search strategy for MEDLINE (EBSCO)^a^.

String number	Search strings	Results, n
String 1	“*ward rounds” OR “rounds”*	90,462
String 2	“*teaching” OR “education” OR “learning”*	1,924,938
String 3 (strings 1 and 2)	*(“ward rounds” OR “rounds”) and (“teaching” OR “education” OR “learning”)*	9316
String 4	“*trainee” OR “junior”*	81,722
String 5 (strings 3 and 4)	*(“ward rounds” OR “rounds”) AND (“teaching” OR “education” OR “learning”) AND (“trainee” OR “junior”)*	579
String 6 (strings 3 and 4)	*(“ward rounds” OR “rounds”) AND (“teaching” OR “education” OR “learning”) AND (“trainee” OR “junior”)*	291

^a^Limiters: scholarly (peer-reviewed) journals, a date of publication ranging from 2015 to 2022, English-language articles, and human studies.

### Inclusion and Exclusion Criteria

Articles addressing trainees’ education in all ward rounds were included. Articles were excluded if they did not relate to education during ward rounds, were specific to certain disciplines, were reviews, were not published in scholarly journals, were published before 2015, were published in languages other than English, or did not concern human participants.

### Data Extraction and Reporting

Two independent reviewers assessed articles for eligibility. In cases of disagreement, discussions were sufficient for reaching an agreement. No third-party review was needed to resolve discrepancies. After excluding articles, the number of remaining relevant articles was reduced by specifying search limiters. Articles were then excluded based on reading the abstracts and titles. A search of the references in the selected articles was also performed. Next, full texts were read to assess articles’ relevance to the selected topic. Afterward, reference searching was performed to identify more articles. Qualitative data on the following themes were extracted: perceptions of the educational value of ward rounds, perceived barriers to teaching and learning, types of educational activities, perceptions of simulation-based ward rounds, the impact of trainee characteristics on the educational experience provided by rounds, and interventions and solutions that were explored to improve learning during ward rounds. The participants’ views and reported outcomes were expressed as percentages.

### Quality Assessment of Studies

The quality of the evidence presented in the selected articles was assessed via the Oxford Centre for Evidence-Based Medicine levels of evidence [7]. The risk of bias within the selected articles was also assessed via the Critical Appraisal Skills Programme (CASP) checklist for qualitative research [8]. For conflicts related to the quality assessment of the studies, a discussion was held between the two authors of this paper, and a final rating was determined. No third party was needed to resolve any conflicts.

## Results

### Search Results

**Figure 1:**
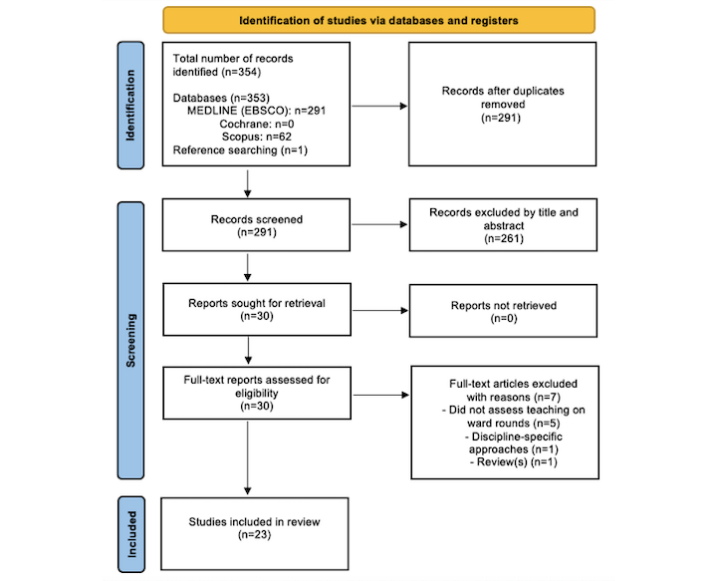
PRISMA (Preferred Reporting Items for Systematic Reviews and Meta-Analyses) flow diagram.

A total of 354 publications were retrieved. Studies were sorted by relevance. After removing duplicates, titles and abstracts were screened to assess eligibility, which resulted in the exclusion of 261 articles. The remaining 30 full-length articles were read, and 7 additional articles were excluded. Exclusion details are outlined in Figure 1. One article was identified by reference searching the selected articles. A total of 23 articles were included in this review (Figure 1 and Table 2).

The themes pertaining to education in ward rounds were identified (Table 3).

**Table 2:** Included studies.

Study (authors, year published)	Study type	OCEBM^a^ evidence level
Gee et al [9], 2015	Audit	2C
Harvey et al [10], 2015	Nonrandomized crossover trial	2B
Laskaratos et al [11], 2015	Cross-sectional observational study	3
Piquette et al [12], 2015	Prospective observational study	2C
Powell et al [13], 2015	Pre-post study	2B
Thomas [14], 2015	Cross-sectional observational study	3
Herrmann et al [15], 2016	Observational study	4
Laskaratos et al [5], 2016	Cross-sectional observational study	3
Rabinowitz et al [16], 2016	Qualitative study	3
Merritt et al [17], 2017	Cross-sectional observational study	3
Beck et al [18], 2018	Prospective observational study	2C
Gray and Enright [19], 2018	Cross-sectional observational study	3
Morgan et al [20], 2018	Pre-post study	2B
Rao et al [21], 2018	Multiarm pre-post study	2C
Somasundram et al [22], 2018	Experimental study	2C
Goodrich et al [23], 2020	Nonrandomized trial	3
Levine et al [24], 2020	Prospective mixed methods	2C
Spence et al [25], 2020	Nonrandomized trial	3
Gray et al [26], 2020	Observational study	4
Armendariz et al [27], 2021	Prospective observational study	2C
Khan et al [28], 2021	Cross-sectional observational study	3
Modak and Gray [29], 2021	Qualitative study	3
Solomon et al [30], 2021	Randomized controlled trial	2B

^a^OCEBM: Oxford Centre for Evidence-Based Medicine.

**Table 3:** Themes pertaining to education during ward rounds in each article.

Articles (authors, year)	Perceptions of the educational value of ward rounds	Perceived barriers to teaching and learning	Types of educational activities	Perceptions of simulation-based ward rounds	Impact of trainee characteristics	Interventions explored to optimize education in ward rounds
Laskaratos et al [5], 2016	✓^a^	✓	✓			
Gee et al [9], 2015				✓		
Harvey et al [10], 2015				✓		
Laskaratos et al [11], 2015	✓	✓				
Piquette et al [12], 2015			✓			
Powell et al [13], 2015				✓		
Thomas [14], 2015				✓		
Herrmann et al [15], 2017						✓
Rabinowitz et al [16], 2016		✓	✓			
Merritt et al [17], 2017)			✓			
Beck et al [18], 2018	✓		✓			
Gray and Enright [19], 2018	✓	✓				
Morgan et al [20], 2018				✓		
Rao et al [21], 2018				✓		
Somasundram et al [22], 2018				✓		
Goodrich et al [23], 2020						✓
Levine et al [24], 2020			✓			
Spence et al [25], 2020				✓		
Gray et al [26], 2020						✓
Armendariz et al [27], 2021		✓				
Khan et al [28], 2021	✓	✓	✓			
Modak and Gray [29], 2020	✓	✓	✓		✓	
Solomon et al [30], 2021						✓

^a^✓: indicates the studies incorporated the relevant theme(s) mentioned in the header.

### Quality Assessment of Studies

**Figure 2:**
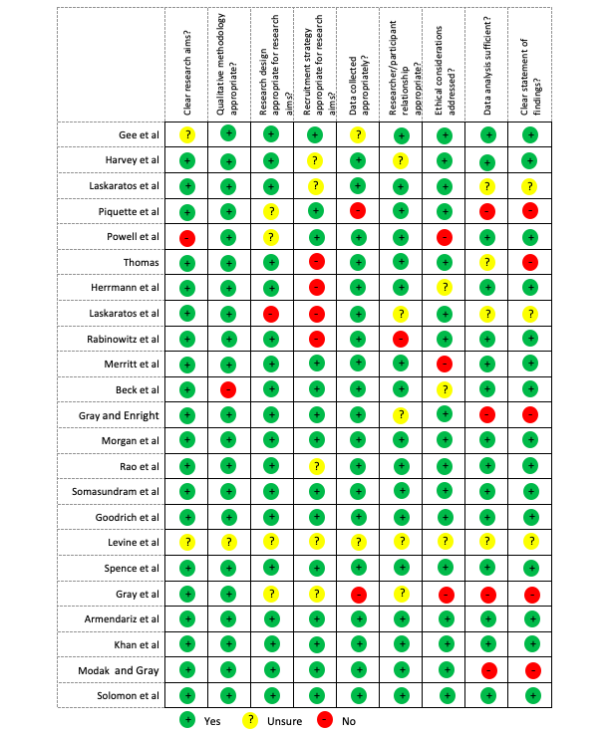
Risk of bias assessment [5,9-30].

Of the 23 included studies, 6 were cross-sectional observational studies, 4 were prospective observational studies, 2 were pre-post studies, 1 was an audit, and 1 was a randomized controlled trial. The details of the other study types can be found in Table 2. The risk of bias was assessed based on the CASP checklist for qualitative research. In general, most papers (19/23, 83%) had a low risk of bias, per our assessment. The findings of this assessment can be found in Figure 2.

### Perceptions of the Educational Value of Ward Rounds

A total of 6 studies assessed perceptions of teaching in rounds [5,11,18,26,28,29] (Table 3). For this review, perceptions of education in rounds were considered positive, ambivalent, and negative if such education was considered acceptable by at least 55% of the respondents, by 50% to 54% of respondents, and by less than 50% of the respondents, respectively. In 3 of the 6 studies, participants had negative perceptions of the educational value of rounds [5,11,18]. Beck et al [18] conducted an observational study that objectively demonstrated that teaching only occurred in 29% of patient encounters. In addition, 2 of the 6 studies reported an ambivalent perception of education. However, Gray and Enright [19] reported a discrepancy between the education received and the education desired by trainees; this study relied on a mixed methods approach, using questionnaire-based perceptions as well as observations. Similarly, Khan et al [28] found that trainees perceived rounds as service oriented and considered them to be “business rounds.” One study reported positive perceptions of educational opportunities among participants. Their perceptions of the educational value of rounds were congruent with consultants’ engagement in education, trainees’ initiative in seeking feedback, and organizational constraints [29].

### Perceived Barriers to Teaching and Learning

A total of 7 studies assessed perceived barriers to teaching during rounds [5,11,16,19,27-29]. Textbox 1 provides details on the perceived barriers in each study. There were many barriers mentioned; however, a recurring theme was noted—time constraints [11,16,28,29]. Although ward rounds are recognized as educational opportunities, teaching during rounds may increase the time needed to conduct rounds. Moreover, interruptions [11,27] have been noted as another common barrier to effective ward rounds. The potential educational aspect of ward rounds is why they are beneficial to trainees; however, it has been noted multiple times that the service-oriented nature of rounds [5,11,28] diminishes this educational element.

**Perceived barriers to teaching and learning during rounds.**

**Laskaratos et al [11], 2015**

- Time constraints
- Workload
- Interruptions
- Service-oriented nature of rounds (competing administrative tasks)
- Sparsity of feedback

**Laskaratos et al [5], 2016**

- Service-oriented nature of rounds

**Gray and Enright [19], 2018**

- Hierarchal nature of rounds (consultant-led rounds)
- Limited opportunities for trainees to be involved in setting management plans
- Limited opportunities to ask questions

**Khan et al [28], 2021**

- Time constraints
- The number of patients (workload)
- Service-oriented rounds

**Modak and Gray [29], 2020**

- Time restrictions
- Workload
- Schedules
- Clerical duties
- Hesitancy to provide feedback
- Consultants’ willingness to teach
- Trainees’ initiative to ask for feedback

**Armendariz et al [27], 2021**

- Interruptions during rounds (eg, interruptions by nurses, consultants, and workers from other disciplines; phone calls; and personal interruptions)

**Rabinowitz et al [16], 2016**

- Time restraints
- Limited working hours
- Variations in participants’ needs

### Impact of Trainee Characteristics

One study addressed the impact of trainees’ characteristics on their perceptions of the educational value of rounds. Modak and Gray [29] found that trainees’ traits influenced their learning; these included the initiative to seek feedback, ask consultants questions, and self-reflect. However, trainees’ willingness to take initiative was influenced by their concerns about consultants’ expectations for their knowledge. Furthermore, the trainees’ states also impacted their learning; the trainees’ states reflected their uncertainty about their knowledge baseline and clinical assessments, as well as their preexisting workloads (clerical responsibilities) and cognitive overload. In addition to the trainees, some registrars were also unwilling to teach residents due to their lack of confidence in their knowledge.

### Types of Educational Activities

A total of 8 studies mentioned learning activities during ward rounds [5,12,17,18,24,28,29] (Table 3). In Laskaratos et al’s [5] 2016 study, senior trainees found rounds useful for learning higher-order skills, such as difficult decision-making. However, senior trainees thought that there were limited opportunities to learn about clinical assessments and gain medical knowledge. Beck et al’s [18] study emphasized medical discussions during rounds as a method of teaching through modeling and observation. The most discussed topic was “cancelling low-value laboratory investigations, therapies, or limiting parameters monitored,” followed by “developing a patient-centered plan.” These topics were discussed in 8% and 7% of encounters, respectively. Merritt et al [17] examined the teaching methods that have been used during rounds and ranked physicians as teachers. Physicians with better teaching ratings performed more patient-specific teaching, discussed general medical topics, and provided trainees with feedback. In Khan et al’s [28] study, participants identified the elements that can be best learned during rounds in descending order, as follows: investigation, management, patient history taking, and patient examination. Furthermore, Modak and Gray [29] identified different didactic strategies, which included ceasing teaching opportunities, performing case-specific reflections, highlighting important information, teaching while having a casual coffee break following rounds, and having consultants explain their rationales and guide residents in making decisions. Levine et al [24] analyzed teaching points related to patient safety; the points were taught through verbal conversations pertaining to inpatient and discharge safety, diagnostic safety and the prevention of errors, medication and procedure safety, communication, and hospital-acquired infections. The verbal patient safety messages were presented as statements (medical orders), inquiries, or factual reaffirmations and reminders. Additionally, Piquette et al [12] explored 2 approaches for teaching in rounds. The first approach was “in series” teaching, which involved a structured session that focused on education that was not interrupted by providing care to patients, whereas the second approach involved highlighting quick learning points for residents “in parallel” with caring for a patient. The advantages of the “in series” approach included providing structured teaching and an opportunity to focus on trainees’ individual learning needs, whereas the “in parallel” approach allowed the educational focus to revolve around the cases encountered. Finally, in a study by Rabinowitz et al [16], residents identified different learning points from rounds, as follows: deriving differential diagnoses and management plans, conducting physical examinations, practicing presenting patients’ conditions to colleagues, communicating plans to patients, and understanding the importance of professionalism.

### Perceptions of Simulation-Based Ward Rounds

Perceptions of learning through simulated ward rounds were assessed by 8 studies [9,10,13,14,20-22,25] (Table 3). All studies reported good perceptions and a general consensus of satisfaction with simulated ward rounds among learners. The use of simulation was useful in highlighting skills that learners can improve [10,14,21,22,25], as well as improving learners’ perceived preparedness and confidence [9,13,14,20,25]. Spence et al [25] reported that participants noticed that simulation sessions helped them recognize the importance of clinical handover and improve their communication, situational awareness, teamwork skills, and ability to make decisions. Furthermore, simulation-based practice has been proposed to train residents when novel round approaches, such as family-centered rounds, are used in some pediatric departments [21].

### Interventions Explored to Optimize Education in Ward Rounds

A total of 4 studies addressed different interventions that were attempted to optimize residents’ educational experiences during ward rounds [15,23,26,30]. Of these, 2 studies attempted to use frameworks that involved the following four stages: planning, implementing a teaching strategy (this can involve asking questions, prompting reasoning, identifying themes, encouraging evidence-based learning, and observing), observing, and ending sessions by reinforcing learning points and participants’ understanding. The aforementioned frameworks were referred to as “set, target, inspect and close” by Gray et al [26] and “plan, do, study, act cycles” by Herrmann et al [15]. The teaching strategy explored by Herrmann et al [15] involved incorporating a clinical librarian in rounds to promote information seeking and encourage trainees to raise relevant clinical questions and use evidence-based practices. These frameworks were well perceived by consultants and rendered educational activities that were explicit and engaging for team members. These strategies were also advantageous because they were congruent with usual rounds and did not consume additional time [15,26]. Furthermore, 2 of the 4 studies investigated the impact of changing the setting of ward rounds on residents’ educational experiences [23,30]. Solomon et al [30] compared rounds conducted at patients’ bedsides with hallway rounds; they found that hallway rounds were perceived to be superior in terms of efficacy and the education rendered. Moreover, Goodrich et al [23] compared hallway rounds to a novel “conference room rounding style.” Conference room rounds were rounds that were performed while sitting in a conference room and involved all of the concerned interdisciplinary stakeholders (eg, physicians, nurses, pharmacists, dietitians, and social workers). Goodrich et al [23] found that conference rounds were associated with greater efficacy, education, and family involvement when compared with hallway rounds.

## Discussion

### Overview

Daily ward rounds are conducted to assess patients’ status and progress throughout their hospital stay and devise management plans accordingly. They provide a great opportunity for trainee doctors to learn. However, the educational component of ward rounds remains an underresearched field; this review was conducted to explore the existing research on this topic.

During rounds, attending physicians can highlight elements of clinical assessments, communication, management, general and evidence-based medical knowledge, and the decision-making process. They may explain rationales for selected approaches, emphasize cost-efficient options or alternatives, and tackle patient-centered approaches. They can also involve learners, answer their questions, and provide feedback [17,19].

### Principal Findings

This review showed that trainees’ views on the educational value of rounds are predominantly negative [5,11,18]. Speculations have risen about the impact of trainee characteristics on residents’ educational attainment in ward rounds; Modak and Gray [29] found that trainees’ willingness to seek feedback, ask questions, and self-reflect impacted their learning. However, trainees’ willingness to take such initiatives was impacted by their concerns about not meeting basic knowledge expectations. There are many factors impeding the teaching process during ward rounds, including time pressures, competing administrative tasks, physicians’ teaching practices, and the consultant-led hierarchal structure of rounds [5,19]. To address time restrictions, Eraut [31] proposed using didactic approaches that are reactive in nature and pertain to the educational encounters that occur at the workplace. Another approach to teaching at the workplace was proposed by Hoffman et al [32], who presented the following options: reflecting during the encounter itself (“reflection-in-action”) and reflecting after the encounter (“reflection-on-action”). Theoretically, considering these options can help learners and educators overcome the system-related barriers to education during rounds. Moreover, simulated ward rounds also present alternate learning opportunities. These simulations have been shown to increase confidence, preparedness, and the awareness of potential hospital-based challenges among learners [9,10,13,14,20]. Other interventions have also been explored, including using frameworks that involve planning, doing activities, receiving feedback, and identifying learning points. In 2 studies, the use of frameworks helped consultants incorporate teaching into rounds and not consume additional time that could have interfered with their schedules [15,26]. Furthermore, a study that compared residents’ educational experiences in bedside rounds to those experiences in hallway rounds found that hallway rounds were associated with better learning experiences [30]. Another study compared hallway rounds to conference rounds. Conference rounds were conducted while sitting in a conference room; they were multidisciplinary rounds that involved a clinical librarian to encourage evidence-based learning. Conference rounds had a higher degree of efficacy and provided a better educational experience [23].

### Comparison to Prior Work

Over the years, the educational value of teaching at the bedside has been commended, as it has been linked to increased information retention [33]; a better understanding of individualized patient management [34]; and more precise differential diagnoses following clinical assessments, which result in fewer unnecessary services [34,35]. The types of educational activities that have been performed at patients’ bedsides include activities for eliciting physical findings based on patient histories and physical examinations [33,34], demonstrating skills under supervision [36], and enhancing communication skills as well as professionalism [34]. Moreover, older studies identified barriers to bedside teaching, such as time constraints, the fear of causing discomfort to patients, distractions, [33,34,36], obstacles to infection control, increased reliance on investigations [34], and the lack of educator training for physicians [35]. Some solutions that were previously proposed by the literature are preparing patients and trainees prior to commencing rounds, assessing junior trainees’ educational needs, changing clinicians’ attitudes toward teaching, and allocating educational tasks among team members [33,36,37].

An article by Kim et al [38] compiled different educational strategies that can be used to improve bedside teaching; 4 of these strategies can be applied to ward rounds. The first strategy was creating a learning culture through role modeling by more senior physicians, rewarding teaching, and encouraging teaching by nurturing leadership development skills. As a part of role modeling, more senior physicians can role model features of humility, the acknowledgement of knowledge deficiencies, the act of asking colleagues for help, self-correction, and the act of apologizing when a mistake is made. The second strategy Kim et al [38] suggested was scaffolding, which means providing trainees with the assistance they need to perform a task and gradually withdrawing the assistance provided until trainees no longer need help performing the task. The third suggested strategy was using the 1-minute preceptor model, which is composed of the following components: committing to a diagnosis, providing reasoning, providing feedback about what was done well, and guiding learners on how to handle mistakes and omissions. Through this model, a teacher can tailor teaching content to the gaps identified in learners’ approaches [38,39]. The fourth strategy that can be used is identifying learning points from encounters and encouraging further reading on these points by assigning short, casual, 5-minute mini-presentations [38]. Another article by Ratnani et al [40] emphasized the importance of bridging the gap between superficial book-learned knowledge and conceptual practical knowledge. Some of the strategies they brought up were aiming to gradually build up trainees’ knowledge, simplifying principles, simplifying knowledge, and comparing and contrasting knowledge.

It is important to recognize ward rounds as missed educational opportunities. This could be addressed by setting plans to incorporate teaching during rounds to make them more learner centered. This would involve physicians planning to engage trainees in the assessment and management of patients while also providing trainees with feedback. This can be followed up by assessing learners’ perceptions of the teaching methods used and the aspects they find most useful via anonymous surveys or quality assessment audits.

### Strengths and Limitations

One of the strengths of this review is that it sought the most recent information on teaching during ward rounds. This is because trainees’ current perspectives would not be masked by the changes in the educational content of ward rounds over time. Furthermore, this review addresses an underresearched topic in the literature and highlights this topic’s importance. This review also has limitations. The content that this review assesses, which includes perceptions, is mainly qualitative in nature, and some of the included articles derived results from a qualitative synthesis, which is suboptimal. Furthermore, there has been no aggregation or standardization for the educational composition of ward rounds, which made the articles difficult to compare. Finally, many of the interventions that have been attempted have not been studied further to adequately assess their efficacy and reliability.

### Conclusion

Despite the potential that ward rounds demonstrate for providing education, learning opportunities are often missed. In many articles (3/6, 50%), trainees perceived rounds to have low educational value. The perceived barriers to teaching during rounds were time constraints and the hierarchal structure and service-oriented nature of rounds. However, simulated ward rounds have been associated with improvements in the confidence and preparedness of learners. There is a need to develop plans that incorporate teaching regularly during ward rounds in the inpatient setting.

## Abbreviations

CASP: Critical Appraisal Skills Programme
PRISMA: Preferred Reporting Items for Systematic Reviews and Meta-Analyses
